# Sub-Cutaneous Injection of Quinine for the Cure of Intermittent Fever

**Published:** 1864-07

**Authors:** Ignatius Langer

**Affiliations:** Davenport, Iowa


ARTICLE XXX.

### SUB-CUTANEOUS INJECTION OF QUININE FOR THE CURE OF INTERMITTENT FEVER.—WHO IS ENTITLED TO PRIORITY IN THE PRACTICE?

PROF. N. S. DAVIS, *Editor Chicago Medical Examiner:*

SIR—Enclosed you will be pleased to find $2.00 for the *Medical Examiner* of 1864.

In the November number, 1863, of the *Medical Examiner*, page 583, I find an article reprinted from the London *Lancet*, penned by W. J. Moore, in Bombay Medical Service, dated Bombay, 1863, wherein he states: "and I have latterly employed a strong solution of quinine for the cure of intermittent fever by the method of sub-cutaneous injection." He is desirous in consequence of the success which has attended the practice to call the attention to this "novel mode of using quinine;" further he says, "since the period I commenced to use quinine in this manner, I have been surprised and pleased to find in one of the Medical Periodicals that the same plan has been pursued by Dr. Chasseaud, of Smyrna, who reports 150 cures, and especially recommends the system in fever complicated with gastric symptoms, when the exhibition of quinine by the mouth is often inefficient, difficult, and hazardous." You will please to recollect that in your presence, at the 12th annual meeting of the American Medical Association, held in Louisville, Ky., in May, 1859, I read a paper on "Sub-cutaneous Injections of medicines in general, and especially of preparations of Peruvian bark in intermittent fevers and other diseases."

At the same meeting a committee was appointed to report at the next meeting. See transactions of the 12th annual meeting of the American Medical Association. By this the profession has evidence of the fact that more than *four years* prior to any notice of Dr. Chasseaud, of Smyrna, and Moore, of Bombay, I had published and circulated my paper on sub-cutaneous injections of medicines in general, and especially of preparations of Peruvian bark in intermittent fevers, and other diseases, and that nearly a year prior to my publication I had practiced it with the best of success; also, that a lengthy communication of twenty-four columns on *hypodermic medication, and cases thus treated mostly with a solution of sulphate of quinine*, was published in the New York *Medical Press*, of June 16, 1860, Vol. III, No. 25, then edited by Drs. Kiernan and O'Meagher, now surgeons in the U.S.V. Army.

I have waited six months, patiently, to see at least those medical papers who were cognizant of my articles in 1859-1860, and who published, the articles mentioned above, in the *London Lancet*, 1863, do justice, if not to me at least to the history of medicine, and to themselves, by either correcting the statement "THIS NOVEL (1863) MODE" *of using quinine*, or by stating by whom and when the first known use was made, and the first public notice was given. Please insert this in the May number, with such comments as you see proper.

Yours, with sincerity,

DR. IGNATIUS LANGER.

*Davenport, Iowa, April* 26, 1864.

